# An overview of health issues and development in a large clinical cohort of children with Angelman syndrome

**DOI:** 10.1002/ajmg.a.61382

**Published:** 2019-11-15

**Authors:** Karen G. C. B. Bindels‐de Heus, Sabine E. Mous, Maartje ten Hooven‐Radstaake, Bianca M. van Iperen‐Kolk, Cindy Navis, André B. Rietman, Leontine W. ten Hoopen, Alice S. Brooks, ENCORE Expertise Center for AS, Ype Elgersma, Henriëtte A. Moll, Marie‐Claire Y. de Wit

**Affiliations:** ^1^ Department of Pediatrics Erasmus MC Rotterdam The Netherlands; ^2^ ENCORE Expertise Center for Neurodevelopmental Disorders Erasmus MC Rotterdam The Netherlands; ^3^ Department of Child‐ and Adolescent Psychiatry and Psychology Erasmus MC Rotterdam The Netherlands; ^4^ Department of Physical Therapy Erasmus MC Rotterdam The Netherlands; ^5^ Department of ENT (Speech & Language Pathology) Erasmus MC Rotterdam The Netherlands; ^6^ Department of Clinical Genetics Erasmus MC Rotterdam The Netherlands; ^7^ Department of Neuroscience Erasmus MC Rotterdam The Netherlands; ^8^ Department of Neurology and Pediatric Neurology Erasmus MC Rotterdam The Netherlands

**Keywords:** development, epilepsy, genotype–phenotype, growth, UBE3A

## Abstract

This study presents a broad overview of health issues and psychomotor development of 100 children with Angelman syndrome (AS), seen at the ENCORE Expertise Center for AS in Rotterdam, the Netherlands. We aimed to further delineate the phenotype of AS, to evaluate the association of the phenotype with genotype and other determinants such as epilepsy and to get insight in possible targets for intervention. We confirmed the presence of a more severe phenotype in the 15q11.2‐q13 deletion subtype. Novel findings were an association of (early onset of) epilepsy with a negative effect on development, a high occurrence of nonconvulsive status epilepticus, a high rate of crouch gait in the older children with risk of deterioration of mobility, a relatively low occurrence of microcephaly, a higher mean weight for height in all genetic subtypes with a significant higher mean in the nondeletion children, and a high occurrence of hyperphagia across all genetic subtypes. Natural history data are needed to design future trials. With this large clinical cohort with structured prospective and multidisciplinary follow‐up, we provide unbiased data on AS to support further intervention studies to optimize outcome and quality of life of children with AS and their family.

## 1 INTRODUCTION

Angelman syndrome (AS) is a rare neurodevelopmental disorder characterized by typical facial features, postnatal microcephaly, severe developmental delay with lack of speech, movement disorders, characteristic behavioral features, a high prevalence of epilepsy, and sleeping and feeding problems. These features are part of the Category A (100%), B (>80%), and C (20–80%) clinical criteria for AS (Williams et al., 2006). AS is caused by mutations affecting the maternally inherited UBE3A gene, most commonly due to a large deletion of the chromosome 15q11.2‐q13 region and in the other cases due to paternal uniparental disomy (UPD), imprinting center defect (IC), or a pathogenic variant of the maternal copy of the UBE3A gene (Beygo et al., 2019; Kishino, Lalande, & Wagstaff, 1997; Matsuura et al., 1997; Williams et al., 2006). Among the children with a 15q11.2–q13 deletion, most have a 5.9 Mb (Class I) or a smaller 5.0 Mb (Class II) deletion (Beygo et al., 2019; Mertz et al., 2013; Tan et al., 2011).

To improve care for AS patients and to acquire more knowledge about the syndrome a multidisciplinary Expertise Center for AS was established in 2010 at the Erasmus MC Sophia Children's Hospital in Rotterdam, the Netherlands as part of the ENCORE Expertise Center for Neurodevelopmental Disorders. The center was founded in close collaboration with both the patient organization and patient advocacy groups. Up until 2017, 100 children have visited the Expertise Center for AS at least once.

Considering the potential upcoming treatments in AS, it is important to get a more detailed view of all the health issues in AS, with a focus on epilepsy and neurodevelopmental outcomes as these are likely to be the target of future interventions (Beaudet & Meng, 2016; Tan & Bird, 2016). Previous studies presented data of children with AS on specific aspects like epilepsy, development, or growth and mainly collected in a research setting (Gentile et al., 2010; Granild Bie Mertz, Christensen, Vogel, Hertz, & Ostergaard, 2016; Mertz, Thaulov, et al., 2014; Tan et al., 2011; Thibert et al., 2009; Thibert, Larson, Hsieh, Raby, & Thiele, 2013). Our patients are taken care of by a multidisciplinary team of specialists, covering all aspects of AS. Based on the incidence of AS (Mertz et al., 2013) and birth data in the Netherlands (Statistics Netherlands' database [Centraal Bureau voor de Statistiek], n.d.), our clinical cohort represents approximately 75% of all children with AS in the Netherlands, forming a relatively unbiased population. The aim of this study was to get a broad overview of health issues and development of our patients and to analyze these issues in relation to their genotype and other potential determinants such as epilepsy. This allows us to further delineate the phenotype of AS, identify opportunities to improve clinical care, better inform parents and to establish natural history data for future intervention studies.

## 2 MATERIALS AND METHODS

This study presents prospectively collected data of the first 100 children with AS who visited our Expertise Center between 2010 and 2017. One other patient was excluded from this cohort due to mosaicism of a deletion of the chromosome 15q11.2‐q13 region and with a far better phenotype (only a mild developmental delay) considered as not comparable with the children with a true deletion. Clinical diagnosis was molecularly confirmed in all children by the referring clinicians with the available genetic techniques at the time of diagnosis. Children were diagnosed with combinations of methylation sensitive digestion, methylation‐specific PCR (MS‐PCR), methylation‐specific multiplex ligation‐dependent probe amplification (MS‐MLPA), microsatellite marker analysis (MSA), fluorescent in situ hybridization (FISH), MLPA analysis of copy number, Comparative Genomic Hybridization (CGH)‐ or Single Nucleotide Polymorphism (SNP)‐array or single UBE3A gene sequencing.

Clinical follow‐up comprised annual visit of the pediatric neurologist and pediatrician. The first visit was attended by a clinical geneticist as well. A standardized medical history was taken including a parental questionnaire on epilepsy, sleep pattern, milestones, development, behavior, nutrition, gastro‐esophageal reflux (GER), defecation pattern, and a physical exam including growth parameters was performed at every visit. Other specialists were consulted or additional examinations such as laboratory tests, radiological examinations, or electroencephalography (EEG) were performed if needed. Cognitive and behavioral assessment was performed by a psychologist and child‐ and adolescent psychiatrist at the age of 3, 7, 11, and 15 years. From 2014 onwards the program was expanded to include standardized testing of language comprehension and (nonverbal) communication and observation of oral motor function by a speech therapist and motor testing by a pediatric physical therapist at the age of 1, 2, 3, 4, 7, 11, and 15 years. Cognitive, motor, and receptive language development was assessed using the Bayley Scales of Infant and Toddler Development third edition (Bayley‐III; Bayley, 2006). With absence of speech the expressive scale of the Bayley‐III did not contribute. A mobility score adapted to the Gross Motor Function Classification Scale(Palisano, Rosenbaum, Bartlett, & Livingston, 2008) was summarized for analysis to three options: not walking (=1), walking with support (=2), or walking independently (=3). Early onset of epilepsy was defined as onset before the age of 2 years. The effect of epilepsy is considered most profound on brain development in the first 2 years (Berg, Levy, & Testa, 2018).

All statistical analyses were performed using IBM SPSS version 24 (Corp, 2016). Unless stated otherwise, the children with a UPD, IC, and a pathogenic variant of the UBE3A gene were grouped and referred to as “nondeletion” group. Differences between groups were calculated with independent *t*‐tests or one‐way ANOVA for normally distributed continuous data. For categorical data a *χ* ^2^ test was performed; for groups of five or less patients a Fisher's Exact test. For non‐normally distributed data, a Wilcoxon signed rank test (or a Mann Whitney *U* test when appropriate) was used. The mobility score, age of independent walking and the raw scores of the Bayley‐III were analyzed with correction for chronological age. We used the data of the most recent visit to the age of 18 years and complete case analyses were performed. All statistical tests were two‐sided and *p*‐values <.05 were considered statistically significant. This study was approved by the Medical Ethics Committee of the Erasmus MC, the Netherlands (MEC‐2015‐203). Written informed consent was formally waived, as there was no patient burden or privacy concern.

## 3 RESULTS

The data of 50 boys and 50 girls with molecularly confirmed AS are presented in Table 1. In 2017, 77 children were in annual pediatric follow‐up, 11 had made the transition to the adult AS clinic at the age of 19 years, and 12 children revisited our center less regularly. The overall mean age at first visit was 5.7 years (*SD* = 4.8), but over time this shifted toward mostly younger children. The mean age at first visit was 2.5 years (*SD* = 2.3) in 2016. There was no significant difference in age at most recent visit between the deletion and nondeletion group.

**Table 1:** General characteristics according to genotype

	Deletion	Nondeletion	Total
*N*	62	38	100
*UPD*		*16*	
*IC*		*4*	
*UBE3A*		*18*	
Sex (male/female)	34/28	16/22	50/50
*UPD*		*7/9*	
*IC*		*3/1*	
*UBE3A*		*6/12*	
Age of diagnosis (mean in months with *SD*)^a^	22.5 (23.7)	33.8 (21.2)	26.8 (23.2)
*UPD*		*34*.*3* (*28*.*9*)	
*IC*		*39*.*8* (*24*.*2*)	
*UBE3A*		*32*.*1* (*12*.*1*)	
Age at first visit (mean in years with *SD*)	5.31 (5.1)	6.34 (4.2)	5.7 (4.8)
Age at most recent visit (mean in years with *SD*)	8.4 (5.4)	9.5 (5.4)	8.8 (5.3)

Abbreviations: IC, imprinting center defect; UPD, uniparental disomy.

a Deletion versus nondeletion *p* < .05.

### 3.1 Genetics

The genetic diagnoses were a chromosomal 15q11.2‐q13 deletion in 62%, UPD in 16%, IC in 4%, and a pathogenic variant of the UBE3A gene in 18%.

Sex was equally divided for children with a deletion, UPD, and IC, but there were twice as many females in the pathogenic variant of the UBE3A gene group.

Children with a deletion were diagnosed at a significantly younger age than the nondeletion children: mean age 22.5 versus 33.8 months (*p* < .05).

### 3.2 Epilepsy and sleep

Eighty‐two percent of the children were diagnosed with epilepsy, with a significantly higher rate in the deletion group, as presented in Table 2. The mean age of onset of epilepsy was 32 months (*SD* = 24.8) with a significantly younger age of onset in the deletion group.

**Table 2:** Epilepsy characteristics according to genotype

	Deletion	Nondeletion	Total
*N*	62	38	100
Epilepsy,^a^ *n* (% of genetic subgroup)**	57 (92)	25 (66)	82
Age of first seizure, mean in months (*SD*)^b^^,^ *	24 (15.6)	52.9 (31.9)	32 (24.8)
Absence seizures^a^, *n* (% of genetic subgroup)	47 (76)	20 (53)	67
Tonic–clonic seizures^a^, *n* (% of genetic subgroup)	30 (48)	13 (34)	43
Atonic seizures^a^, *n* (% of genetic subgroup)	12 (19)	9 (24)	21
Nonconvulsive status epilepticus, *n* (% of genetic subgroup)*	16 (25)	3 (0.1)	19
Convulsive status epilepticus, *n* (% of genetic subgroup)	5 (0.1)	0 (0)	5
Epilepsy in remission with or without AED^a^, *n* (% of genetic subgroup)	23 (40)	8 (32)	31

Abbreviation: AED, antiepileptic drugs.

a Defined as now or earlier.

b Data from two deletion children missing.

* Deletion versus nondeletion *p* < .05.

** Deletion versus nondeletion *p* < .01.

Most children (67%) had absence seizures. Tonic–clonic seizures were reported in 43%, atonic seizures in 21%. An episode of a nonconvulsive status epilepticus (NCSE) occurred in 19% and a convulsive status epilepticus in 5%. The occurrence of NCSE was significantly higher in the deletion group. Of the 82 children with epilepsy, 38% achieved seizure control on treatment with antiepileptic drugs (AED), 33% were still on, and 5% were successfully withdrawn from AED treatment. Valproic acid was most commonly used as AED (65%), followed by clobazam (33%), levetiracetam (25%), ethosuximide (15%), and clonazepam (14%). Monotherapy was sufficient for 46% of the children, usually this was valproic acid (61%). Valproic acid was discontinued in 20 children because of side effects (50%), lack of efficacy (30%), and/or seizure remission of more than 2 years (25%). Reported side effects were tremor, behavioral problems, drowsiness, nausea, skin rash, and/ or elevated serum ammonia. Six percent of the children with epilepsy were effectively treated with a low glycemic index diet or ketogenic diet.

Sleep problems were reported by 91% of the parents, with difficulty of settling in 57%, frequent waking in 87% and early waking in 59% of the children. There were no genotype differences. Medication to improve sleep was used by 46% of the children. Melatonin was used currently by 34% with variable doses and response and previously by 30% of the children. No association between sleep problems and epilepsy was found (*p* = .16). Almost 20% of the parents reported co‐sleeping.

### 3.3 Growth and other health issues

**Figure 1:**
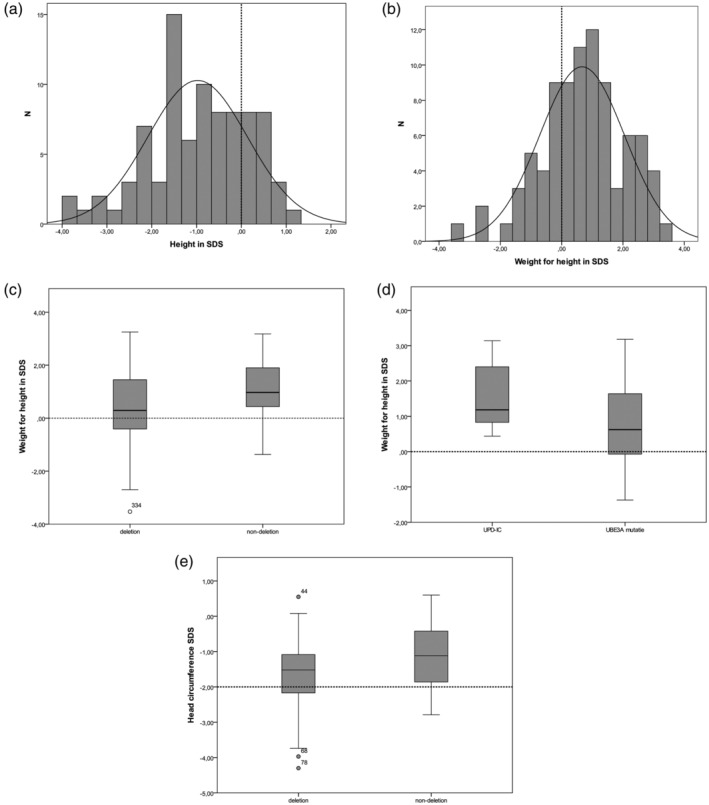
Growth parameters. (a) Mean height, (b) Mean weight for height, (c) Mean weight for height deletion versus nondeletion, (d) Mean weight for height UPD‐IC versus UBE3A‐mutation, (e) Mean head circumference at most recent measurement. Dashed line indicates 0 SDS in (a)–(d) and indicates −2 SDS as demarcation of microcephaly in (e). IC, imprinting center defect; UPD, uniparental disomy

Mean head circumference was −1.4 SDS (*SD* = 1.0) at the most recent measurement and was significantly lower in the deletion group (Table 3 and Figure 1e). Microcephaly (≤−2.0 SDS) at the age of 2 years and older was seen in 24% of all children, twice as many children in the deletion group (30 vs. 15%) and absolutely seen in only five children in the nondeletion group.

**Table 3:** Growth, feeding problems, and other health issues according to genotype

	Deletion	Non‐deletion	Total
*N*	62	38	100
*UPD*		*16*	
*IC*		*4*	
*UBE3A*		*18*	
Head circumference^a^, mean in SDS (*SD*) (*n* = 90)**	−1.67 (1.0)	−1.08 (0.93)	−1.44 (1.02)
Head circumference ≤−2 SDS at the age of 2 years or older, *n* (% of genetic subgroup) (n = 83)	15 (30)	5 (15)	20 (24)
Height, mean SDS (*SD*) (*n* = 86)	−1.1 (1.1)	−0.86 (1.1)	−0.98 (1.1)
*UPD*		*−0*.*7* (*1*.*4*)	
*IC*		*−1*.*0* (*0*.*5*)	
*UBE3A*		*−0*.*9* (*1*.*0*)	
Weight for height, mean in SDS (*SD*) (*n* = 86)*	0.35 (1.5)	1.15 (1.2)	0.67 (1.39)
*UPD*		*1*.*5* (*0*.*9*)	
*IC*		*1*.*2* (*0*.*3*)	
*UBE3A*		*0*.*8* (*1*.*4*)	
Tube feeding,^b^ *n* (% of genetic subgroup)*	12 (19.4)	1 (2.6)	13
*UPD*		*0*	
*IC*		*0*	
*UBE3A*		*1*	
Hyperphagia, *n* (% of genetic subgroup)	16 (26)	16 (42)	32
*UPD*		*7* (*44*)	
*IC*		*2* (*50*)	
*UBE3A*		*7* (*39*)	
GER^b^, *n* (% of genetic subgroup)**	39 (63)	11 (29)	50
Constipation^b^, *n* (% of genetic subgroup)	28 (45)	10 (26)	38
Strabismus, *n* (% of genetic subgroup)	27 (44)	13 (34)	40
Refractive error, *n* (% of genetic subgroup)	24 (39)	11 (29)	35
Scoliosis, *n* (% of genetic subgroup)	14 (23)	4 (10)	18

Abbreviation: GER, gastro‐esophageal reflux.

a At most recent measurement.

b Defined as now or earlier.

* Deletion versus nondeletion *p* < .05.

** Deletion versus nondeletion *p* < .01.

Mean height was −0.98 SDS (*SD* = 1.1) with a left shift of 1 SDS of the normal distribution curve (Figure 1a). No effect of genotype was observed. Being overweight (weight for height ≥1 SDS) was seen in 18% and obesity (weight for height ≥2 SDS) in 20% of the children. The mean weight for height in the total group was 0.67 SDS (*SD* = 1.4) with a right shift of 1 SDS of the normal distribution curve (Figure 1b). The mean weight for height was above 0 SDS in all separate genetic subtype groups. Children in the nondeletion group had a significantly higher mean weight for height than the children with a deletion (Figure 1C). Within the nondeletion group children with a UPD or IC (grouped) had a nominal significant higher weight for height than those with a pathogenic variant of the UBE3A gene (*p* = .05; Figure 1D). No sex difference was observed for weight for height.

Significantly more children with a deletion currently or previously needed tube feeding. Thirty‐two percent of the parents reported hyperphagia, varying from no intrinsic limit in eating to searching for food and eating nonfood items. There was no genotype association with hyperphagia. Weight for height was significantly higher in the children with hyperphagia (*p* < .001).

Gastro‐esophageal reflux was reported significantly more often in the deletion children. GER medication was currently used by 27% and laxatives were used by 39% of the children. There was a trend toward more constipation in the deletion children (*p* = .08). GER was seen in all age groups.

Strabismus was seen in 40% and refraction errors were seen in 35% of the children. There was no significant effect of genotype. Scoliosis was seen in 18% of the children, of whom one needed surgery. There was a trend toward a higher rate of scoliosis in the deletion group (*p* = .10).

### 3.4 Development

**Figure 2:**
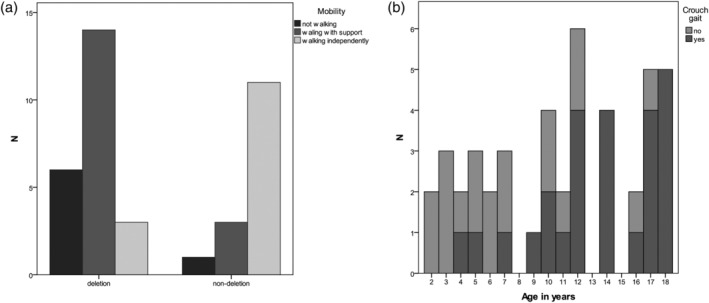
Mobility parameters. (a) Mobility score versus genotype, (b) Crouch gait

Children with a deletion had a significantly lower mobility score, started walking independently later, and walked without support less often than those with a nondeletion (Table 4 and Figure 2a). Age of onset of independently walking was not significantly associated with epilepsy or age of onset of epilepsy. Three children had lost their ability to walk independently. Crouch gait (i.e., walking in hip and knee flexion and toeing out) was observed in 25% of the children. Crouch gait was seen mostly in the older children, mean age of 13.4 years (*SD* = 4.2), as shown in Figure 2b. No association with genotype, epilepsy, weight for height, or scoliosis was found.

**Table 4:** Mobility and motor, cognitive and language development according to genotype

	Deletion	Nondeletion	Total
*N*	62	38	100
Mobility score mean (*SD*) (*n* = 38)**	1.87 (0.63)	2.67 (0.62)	2.29 (0.76)
Independent walking, *n* (% of genetic subgroup)***	25 (40)	30 (79)	55
Age of independent walking (mean in months and *SD*)***	57.9 (29.5)	41.1 (16.1)	48.9 (25.2)
Nonambulatory at age ≥4 years, *n* (% of genetic subgroup)***	24 (39)	2 (0.05)	26
Loss of walking	0	3	3
Crouch gait, *n* (% of genetic subgroup)	15 (24)	10 (26)	25
Bayley GM DQ (*SD*) (*n* = 67)	16.4 (9.9)	19.9 (12.2)	17.6 (11.2)
Bayley FM DQ (*SD*) (*n* = 66)	16.7 (13.0)	23.01 (12.98)	19.0 (13.6)
Bayley cognitive DQ (*SD*) (*n* = 48)*	15.6 (8.3)	23.9 (14.5)	18.0 (11.6)
Bayley receptive language DQ (*SD*) (*n* = 41)**	14.8 (9.4)	26.9 (14.97)	20.6 (13.5)

Abbreviations: Bayley, Bayley scales of infant and toddler development; DQ, developmental quotient; FM, fine motor; GM, gross motor.

* Deletion versus nondeletion *p* < .05.

** Deletion versus nondeletion *p* < .01.

*** Deletion versus nondeletion *p* < .001.

**Figure 3:**
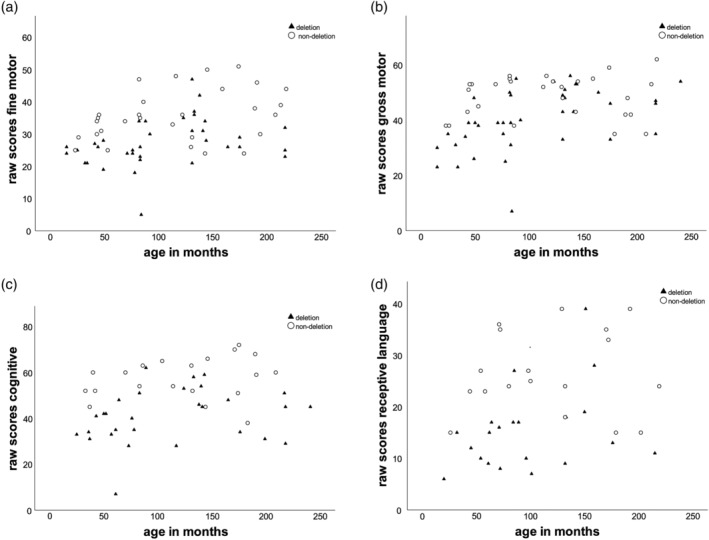
Developmental parameters. (a) Bayley fine motor raw scores versus age, (b) Bayley gros motor raw scores versus age, (c) Bayley cognitive raw scores versus age, (d) Bayley receptive language raw scores versus age

Figure 3 shows the Bayley‐III raw scores of the gross motor, fine motor, cognitive, and receptive language scales as a function of age. The children with a deletion had significantly lower raw scores than those with a nondeletion on both fine and gross motor, cognitive, and receptive language development (all *p* < .05). The developmental quotient (DQ, calculated as developmental age divided by chronological age, times 100) was not different between deletion and nondeletion for gross motor and fine motor development, but significantly different for cognitive and receptive language development (Table 4).

Children with epilepsy had a significantly lower DQ for both gross and fine motor development (both *p* < .01) and cognitive and receptive language development (both *p* < 0.05), as well as significantly lower raw scores on gross motor, cognitive, and receptive language development than those without epilepsy (all *p* < .05). A trend was found toward lower raw scores for fine motor development in the group with epilepsy (*p* = .06). Children with an epilepsy onset before 2 years of age obtained significantly lower raw scores for both gross and fine motor development than children with an onset after 2 years of age (both *p* < .001). A similar association was found for cognitive and receptive language raw scores (both *p* < .01).

## 4 DISCUSSION

This study presents a broad overview of health issues and development and its associations with genotype and epilepsy of the first 100 children with AS seen at our Expertise Center for AS. This is the largest clinical cohort described thus far. Our data confirm the distribution of the genetic subtypes, the overall clinical presentation and the presence of a more severe phenotype in the 15q11.2‐q13 deletion subtype (Clayton‐Smith & Laan, 2003; Luk & Lo, 2016; Mertz et al., 2013; Shaaya, Grocott, Laing, & Thibert, 2016; Tan et al., 2011). Novel findings are a negative association of epilepsy and an earlier age at onset of epilepsy on development, a high occurrence of NCSE, crouch gait in the older children, a relatively low occurrence of microcephaly, a higher mean weight for height in all genetic subtypes with a significant higher mean in the nondeletion children, and a high occurrence of hyperphagia in all genetic subtypes.

### 4.1 Genetics

Overall, the distribution of the underlying genetic causes in our AS cohort is comparable to AS cohorts as reported for the United States, Denmark, and Hong Kong, with chromosomal 15q11.2‐q13 deletion being the most common cause and an IC abnormality showing the lowest prevalence (Clayton‐Smith & Laan, 2003; Luk & Lo, 2016; Mertz et al., 2013; Shaaya et al., 2016; Tan et al., 2011). In our cohort, the mean age of diagnosis was significantly earlier in children with a deletion than in children with a nondeletion, similar to other cohorts (Mertz et al., 2013; Tan et al., 2011). This may reflect both the more severe phenotype and the current availability of micro‐array as a first genetic tool in the diagnostic work‐up of children with a developmental delay. Age of diagnosis for UPD and pathogenic variant of the UBE3A gene was about a year younger in our cohort compared to children diagnosed between 1991 and 2009 in Denmark (Mertz et al., 2013). This may reflect growing awareness of AS since a methylation test and UBE3A gene sequencing test are performed only upon a strong clinical suspicion of AS.

### 4.2 Epilepsy

Up to 90% of the children with AS develop epilepsy and children with a deletion have a higher risk as well as an earlier onset of epilepsy, as was also confirmed in our cohort (Conant, Thibert, & Thiele, 2009; Granild Bie Mertz et al., 2016; Lossie et al., 2001; Pelc, Boyd, Cheron, & Dan, 2008; Tan et al., 2011; Thibert et al., 2009, 2013). A more severe epilepsy phenotype is thought to be associated with the involvement of the GABA‐A receptor subunit cluster, which is part of the 15q11.2‐q13 locus. The occurrence of absence seizures and NCSE in our cohort is high, as was also reported by Worden, Grocott, Tourjee, Chan, and Thibert (2018). Early recognition of NCSE is important, since we often observed a delay of days or weeks in the correct diagnosis and a long time to recover to the pre‐NCSE level of functioning. We recommend educating the parents about the signs of NCSE and to have prompt EEG confirmation in case of NCSE suspicion to start treatment as early as possible. Symptoms of NCSE that were seen in our cohort were lowering of consciousness where patients were still responsive to stimuli, however unusually slow. Also loss of already acquired milestones, less interest in food, increased drooling, and episodes of staring were seen. If children regularly have tonic–clonic or atonic seizures, the frequency of these seizures was much lower during NCSE.

In our study 46% of the patients achieved seizure control using a single AED, which was valproic acid in two‐third of the cases. The majority tolerated valproic acid well, in contrast to recent data from Boston, where only 8% remained on valproic acid because of side effects (Shaaya et al., 2016). In Denmark and Boston benzodiazepines and levetiracetam, respectively, are prescribed as monotherapy most commonly, which highlights local differences in AED treatment preferences (Granild Bie Mertz et al., 2016; Shaaya et al., 2016).

### 4.3 Development

Gross and fine motor, cognitive, and receptive language Bayley‐III raw scores, and cognitive and receptive language DQ were significantly lower in the children with a deletion compared to the nondeletion group, as was published previously (Gentile et al., 2010; Mertz, Thaulov, et al., 2014). Scores for both motor skills and cognitive and language development were also significantly negatively associated with (early onset of) epilepsy. This association has not been reported before. One small study (*n* = 11) did not find an association between epileptic activity and developmental milestones (Ohtsuka et al., 2005). The question is whether (early onset of) epilepsy is a direct cause of more severe developmental delay (e.g., by interfering with brain development) or whether both epilepsy and more severe developmental delay share a common neuropathological etiology. It is unsure whether early or more aggressive treatment of epilepsy in AS contributes to a better outcome, although this has been shown in other syndromes (Curatolo et al., 2018).

The mean age of onset of independent walking and associated genotype difference is comparable to earlier findings (Lossie et al., 2001). Possible factors in the delay or failure of independent walking can be instability due to tremor, abnormal muscle tone, balance problems, epilepsy, and/or visual problems. To our knowledge, the high rate of crouch gait in the older children has not been reported earlier. We did not find an association with sex, genotype, weight for height, nor scoliosis. Lack of muscle strength, abnormal muscle tone, and/or peripheral neuropathy could explain this gait problem. Further research is necessary to gain more insight in the underlying mechanisms of gait problems to seek for ways to prevent or delay crouch gait since it can contribute to deterioration of mobility.

Visual problems were found in 40% of our children, as reported before (Micheletti et al., 2016; Michieletto, Bonanni, & Pensiero, 2011). Visual function is highly important in mobility, motor and cognitive development, and communication, so standard periodic screening is recommended.

### 4.4 Sleep

The high prevalence of sleep problems in AS was confirmed in our cohort and observed for all genotypes (Conant et al., 2009; Spruyt, Braam, & Curfs, 2018; Thibert et al., 2013). Frequent waking occurred most frequently (87%) in our cohort, problems in settling less frequently than previously reported (Conant et al., 2009; Spruyt et al., 2018; Thibert et al., 2013). Possibly less problems in settling are due to the high use of melatonin (in the Netherlands freely available). Whether epilepsy and/or AED cause sleep problems and/or whether sleep deficit increases seizure susceptibility remains uncertain. The earlier reported association between sleeping problems and epilepsy could not be confirmed (Conant et al., 2009). Both biological and behavioral factors may influence sleep in AS. In mouse models a mechanistic connection was found between the lack of UBE3A expression, interaction with clock genes, and changes in sleep–wake cycle (Ehlen et al., 2015; Salminen, Crespi, & Mokkonen, 2019; Shi, Bichell, Ihrie, & Johnson, 2015). Behavioral treatment on the other hand can have a positive effect on sleep problems in AS (Allen, Kuhn, DeHaai, & Wallace, 2013). Sleep problems in children (frequent wakening and if not primarily intended co‐sleeping) usually result in sleep problems for parents as well; parents rate sleep problems as one of their most serious concerns (Grieco, Romero, Flood, Cabo, & Visootsak, 2019; Wheeler, Sacco, & Cabo, 2017).

### 4.5 Growth and other health issues

Disproportionate growth of the head circumference, usually leading to microcephaly (≤−2 SDS) at the age of 2 years and older is part of the B category clinical criteria for AS, which implicates a prevalence of >80% in AS (Williams et al., 2006). Although in our cohort the mean head circumference was below average, with a lower mean in the deletion group, microcephaly was seen in only 24% of all children of 2 years and older and in only five children in the nondeletion group. In previous studies the prevalence of microcephaly varied between 38 and 80% (Mertz, Christensen, et al., 2014; Tan et al., 2011). Our findings and those of the Danish study indicate that microcephaly could be shifted from the B to the C category of AS diagnostic criteria and that absence of microcephaly should not lead to rejection of a clinical suspicion of AS, especially not of the nondeletion type.

Being overweight and obesity had a, respectively, 1.5‐ and 7‐fold higher occurrence in our AS cohort compared to healthy children in the Netherlands (prevalence of 10.5 and 2.8%, respectively [Statistics Netherlands' database (Centraal Bureau voor de Statistiek), n.d.]. This finding was significantly higher in the nondeletion children, as was reported earlier [Brennan et al., 2015; Mertz, Christensen, et al., 2014; Tan et al., 2011]), but the mean weight for height in all genetic subtypes was above 0 SDS. As far as we know, this is a new finding. There was also a difference within the nondeletion group, with UPD and IC children taken together having a nominal significantly higher weight for height than the children with a pathogenic variant of the UBE3A gene. A similar trend was reported in a previous study of, respectively, 24 and 16 nondeletion children (Brennan et al., 2015; Tan et al., 2011), which was attributed to the increased expression levels of paternally expressed genes within the 15q11‐13 locus. However, children with Prader‐Willi syndrome (PWS) also show obesity and carry a maternal UPD at the same locus (Brennan et al., 2015; Mertz, Christensen, et al., 2014). Moreover, also children with a pathogenic variant of the UBE3A gene show a higher weight for height than deletion and non‐AS children. This has also been observed in Ube3a‐mouse mutants, which was shown to be correlated with neuronal CAMK2 activity (van Woerden et al., 2007). Hence, further research is needed to understand the mechanisms leading to weight gain in patients with mutations at the 15q11‐13 locus. Regardless of the molecular mechanism, the metabolic resting expenditure (REE) could be lower in AS, as is seen in PWS (Alsaif et al., 2017). To date, no endocrine studies, skeletal bone age evaluation, nor REE assessments have been published to gain better understanding of growth and metabolism in AS.

One‐third of our cohort showed hyperphagia as was reported previously in children with AS (Barry, Leitner, Clarke, & Einfeld, 2005; Mertz, Christensen, et al., 2014; Salminen et al., 2019; Welham et al., 2015; Williams et al., 2006). In the Danish cohort children with UPD showed significantly more hyperphagia, leading the authors to the hypothesis of a role for paternal gene overexpression, as discussed with the higher weight association (Mertz, Christensen, et al., 2014). However, we identified hyperphagic behavior in all genetic subtypes with no effect of genotype. This discrepancy in findings might be due to the small number of children with UPD and pathogenic variant of the UBE3A gene (*n* = 9) and the absence of children with IC in the Danish study compared to size (*n* = 38) of our nondeletion group (including four children with IC).

The somewhat lower mean height is difficult to interpret, as parental height data were partly missing. Genotype differences were not found, in contrast to previous studies, but information about target height was lacking in these studies as well (Mertz, Christensen, et al., 2014; Tan et al., 2011).

Only a small number (13%) of our children needed tube feeding and nearly all of them carried a deletion. This rate and genotype association is similar to a recent study from the Boston group (Glassman et al., 2017). More severe oral motor problems and general hypotonia probably play a role in this genotype difference. The occurrence of GER, constipation, and scoliosis was comparable to previous reports (Glassman et al., 2017; Sachdeva, Donkers, & Kim, 2016).

### 4.6 Strengths and limitations

An important strength of this study is that, to our knowledge, it presents the largest clinical AS cohort, with prospectively collecting information of both health issues and development. This cohort represents approximately 75% of all children with AS in the Netherlands, which limits referral bias. Most issues regarding health and development (epilepsy, sleep, gastrointestinal problems, mobility, cognition, communication, and behavior) that are addressed in our clinical follow‐up were recently stated as being very important concerns for parents of children with AS (Grieco et al., 2019; Wheeler et al., 2017) and will be targeted as relevant clinical outcomes in future trials.

Limitations of this study are that children had different ages at referral. Some information like achieved milestones from before referral had to be collected retrospectively with risk of recall bias. The average age of the first visit is much lower currently due to referral immediately after diagnosis, which enables us to register data in greater detail and also study age effects in the future. We had some missing data in our standardized routine data, which reduced the power for certain analyses and may have led to an under‐ or overestimation of associations. We were not able to analyze possible differences within the deletion group based on deletion size. Previous studies (Gentile et al., 2010; Mertz, Thaulov, et al., 2014) have not shown differences between Class I and II with respect to development, but it would be interesting to repeat these analyses in a larger sample size. A final limitation is the small group of IC patients (*n* = 4). In order to have sufficient statistical power, we grouped them with the children with a UPD and a pathogenic variant of the UBE3A gene. It would be of great interest to investigate larger numbers of these subgroups in the future.

## 5 CONCLUSION

Overall, the global phenotype of these 100 AS children is in line with previous studies including the more severe phenotype in the 15q11.2‐q13 deletion subtype. Novel findings in our cohort are the association of (early onset of) epilepsy with a less favorable outcome regarding motor, cognitive, and receptive language development. Possibly, early epilepsy treatment may improve outcome. Furthermore, we found a not previously reported high rate of crouch gait in the older children with a risk of deterioration of mobility, a relatively low occurrence of microcephaly, a higher mean weight for height in all genetic subtypes with a significant higher mean in the nondeletion children, and a high occurrence of hyperphagia in all genetic subtypes.

In general, only limited data are available regarding underlying mechanisms of epilepsy, sleep problems, development delay, gait problems, hyperphagia, and growth, needing further translational research to seek for better understanding and intervention possibilities. With this large clinical cohort with structured prospective and multidisciplinary follow‐up, our ENCORE Expertise Center for AS has the baseline data available to evaluate the effect of potential treatment modalities on relevant clinical outcomes.

### 5.1 ENCORE Expertise Center for AS

Philine Affourtit, Karen G. C. B. Bindels‐de Heus, Alice S. Brooks, Hennie T. Brüggenwirth, Gwen C. Dieleman, Bram Dierckx, Ype Elgersma, Leontine W. ten Hoopen, Maartje ten Hooven‐Radstaake, Bianca M. van Iperen‐Kolk, Jeroen S. Legerstee, Elles J.T.M. van der Louw, Henriëtte A. Moll, Sabine E. Mous, Cindy Navis, Pieter F.A. de Nijs, André B. Rietman, Marlies J. Valstar, Marie‐Claire Y. de Wit.

## CONFLICT OF INTEREST

None.

## Data Availability

The data that support the findings of this study are available from the corresponding author upon reasonable request within national legal bounds.
